# A Bayesian approach to modeling antimicrobial multidrug resistance

**DOI:** 10.1371/journal.pone.0261528

**Published:** 2021-12-29

**Authors:** Min Zhang, Chong Wang, Annette O’Connor

**Affiliations:** 1 Department of Statistics, Iowa State University, Ames, Iowa, United States of America; 2 Department of Veterinary Diagnostic and Production Animal Medicine, Iowa State University, Ames, Iowa, United States of America; 3 Department of Large Animal Clinical Sciences, Michigan State University, East Lansing, Michigan, United States of America; University of Illinois College of Veterinary Medicine, UNITED STATES

## Abstract

Multidrug resistance (MDR) has been a significant threat to public health and effective treatment of bacterial infections. Current identification of MDR is primarily based upon the large proportions of isolates resistant to multiple antibiotics simultaneously, and therefore is a belated evaluation. For bacteria with MDR, we expect to see strong correlations in both the quantitative minimum inhibitory concentration (MIC) and the binary susceptibility as classified by the pre-determined breakpoints. Being able to detect correlations from these two perspectives allows us to find multidrug resistant bacteria proactively. In this paper, we provide a Bayesian framework that estimates the resistance level jointly for antibiotics belonging to different classes with a Gaussian mixture model, where the correlation in the latent MIC can be inferred from the Gaussian parameters and the correlation in binary susceptibility can be inferred from the mixing weights. By augmenting the laboratory measurement with the latent MIC variable to account for the censored data, and by adopting the latent class variable to represent the MIC components, our model was shown to be accurate and robust compared with the current assessment of correlations. Applying the model to *Salmonella* heidelberg samples isolated from human participants in National Antimicrobial Resistance Monitoring System (NARMS) provides us with signs of joint resistance to Amoxicillin-clavulanic acid & Cephalothin and joint resistance to Ampicillin & Cephalothin. Large correlations estimated from our model could serve as a timely tool for early detection of MDR, and hence a signal for clinical intervention.

## Introduction

### Background

The phrase, multidrug-resistant isolate, is typically used to refer to a bacterial isolate that is resistant to at least one antibiotic in three or more drug classes. The use of antibiotics for bacterial diseases of human and animals contributes to the selection of pathogenic bacteria resistance to multiple drugs [1]. In fact, multidrug resistance has become a significant threat to public health and the effective treatment of bacterial infections. Research has shown that the in-hospital costs attributable to multidrug resistance isolates are alarmingly high, justifying the application of strict infection control measures in medical institutions with increased rate of multidrug-resistant infections [2]. It was also concluded by Giamarellos-Bourboulis et al. (2006) [3] that patients with infections by multidrug-resistant isolates were associated with decreased survival compared with infection by susceptible isolates.

**Fig 1:**
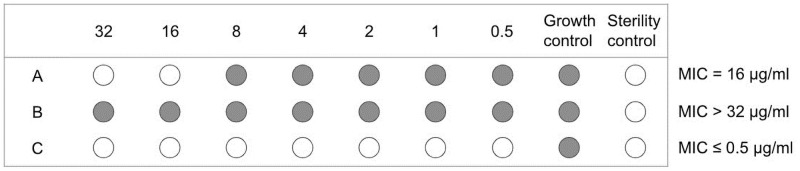
Schematic of the microtiter plate for broth microdilution experiment to determine the minimal inhibitory concentration (MIC) [5]. A: MIC is recorded as = the lowest concentration that inhibits visible bacterial growth; B: MIC is recorded as > the highest concentration when growth occurs in all dilutions; C: MIC is recorded as ≤ the lowest concentration when no growth occurs in any concentrations.

In order to promote and protect public health by providing information about emerging bacterial resistance, resistance spreading pattern, and the impact of interventions, the National Antimicrobial Resistance Monitoring System (NARMS) was established in 1996. NARMS is a collaborative effort of three federal agencies: the Centers for Disease Control and Prevention (CDC), the U.S. Food and Drug Administration (FDA), and the United States Department of Agriculture (USDA), as well as state and local health departments in all 50 states [4]. NARMS tests isolates to determine their antibiotic susceptibility by finding the minimum inhibitory concentration (MIC, expressed in μg/mL), which is defined as the lowest concentration of a particular antibiotic that inhibits the visible growth of the bacteria. To date, dilution experiments [5] and multiple computational approaches (e.g., whole-genome sequencing based methods [6–9]) can be carried out for MIC determination. In NARMS, broth microdilution, as shown in Fig 1, on the Sensititre System from Trek Diagnostics was the method used to determine MICs for isolates [10]. Relative to the MIC breakpoints adopted from the Clinical and Laboratory Standards Institute (CLSI), isolates are classified as susceptible, intermediate, and resistant.

With societies investment in large-scale surveillance systems like NARMS, it is critical that we obtain as much value from the collected data as possible. Because multidrug resistance is particularly important, it would be desirable if the trends could be evaluated from multiple aspects when antibiotics appear to be jointly acquiring resistance. For example, knowledge that both drug A and drug B were showing increasing MIC levels in the same population of bacteria could trigger an investigation into why and possible early mitigation. Currently, this simultaneous increase in MIC of drug A and drug B would be detected only if both drugs increased resistance sufficiently to cause a change in the percentage of resistant bacteria. Such an approach to detection is very limited in scope. What is needed is a method that provides a more sensitive signal to public health officials of emerging trends that can trigger closer scrutiny earlier. For example, if both drug A and drug B have increasing MICs below the breakpoint threshold (or above the threshold) then current methods could not detect this change. Similarly, if resistance is increasing in two drugs, but only one at a level that changes organisms from being sensitive to resistant, current analysis methods could not detect this important joint emergence of resistance either. Therefore, there is a need to develop methods of detecting correlations in changes in MICs as well as changes in resistance category, which allows detection of joint resistance emergence in a more timely manner using already collected data.

### Literature review

In practice, MIC data are often dichotomized to resistant and non-resistant and isolates with intermediate susceptibilities are often considered as susceptible [11]. A predominant approach to assessing the antimicrobial resistance (AMR) has focused on the proportion of resistant component, which can be found in research about AMR temporal trends [12, 13], cross-population correlation in AMR [14, 15], and multidrug resistance (MDR), etc.

For research in MDR, a survey on *Mannheimia* haemolytica isolated from respiratory diseased bovine shows an increasing pattern of antimicrobial MDR from 42% to 63% between 2009 and 2011 simply using descriptive statistics [16]. They also conducted mixed-effect logistic regression to analyze coresistance, i.e. the probability that resistance to a given agent was associated with resistance to at least one other antimicrobial. More examples of using descriptive statistics to demonstrate MDR are found in the NARMS final report [10], as well as MDR studies in *Salmonella* isolates from humans in France [17] and Taiwan [18], etc. To examine and quantify the temporal trends of multidrug resistant gram-negative bacilli, resistant proportion data from a nine-year nosocomial surveillance study was analyzed using the chi-square test for trend, from which a rapid increasing pattern of resistance to three or more antimicrobials was discovered [19].

Another approach to revealing MDR was to calculate the correlation between resistance to one drug and resistance to one or more other drugs within a bacterial population. The correlation can be calculated using data of binary classification of isolates’ susceptibility (resistant v.s. non-resistant) or the quantitative MICs of several antibiotics [20, 21]. In an analysis of MDR in the European Union (EU), the multivariate binary resistance patterns were modeled with a generalized linear model whose within-subject correlation matrix defines the dependence among multiple antimicrobials [22]. The model parameters were estimated using a generalized estimating equations (GEE) approach, which led to the same correlation estimation as the Spearman rank correlation. All of the above-mentioned research articles on MDR were based upon MIC dichotomization defined by CLSI and the observed MIC values.

Methods based on categorization of quantitative data can cause information loss [23]. The CLSI breakpoints for several antibiotics have gone through changes during the NARMS program, leaving it inappropriate to compare of resistant proportions over time. Additionally, direct calculation with the observed MIC values could also be problematic, as they are all subject to the censoring issue. The observed “MIC = 16 μg/mL” of organism A in Fig 1 is interval censored, which actually indicates that the true MIC is >8 and ≤ 16 μg/mL” but ultimately unknown. Similarly, organism B (MIC > 32 μg/mL”) and C (MIC ≤ 0.5 μg/mL”) are right and left censored at the highest and lowest concentration of the serial dilution experiment, respectively. Therefore, what we actually observe from the MIC measurement is the interval where the true MIC lies in. Correlation in MIC between antibiotics could possibly be overestimated or underestimated if not adjusting for the censored nature of the data.

As part of detecting trends in increasing resistance in several antibiotics concurrently, it would be helpful to have an estimation on the correlation in the resistance level among different drugs, which includes two interesting perspectives:

i. Correlation in the continuous latent MICs, meaning that high MIC values to one drug indicate high MIC values to the other(s).
ii. Correlation in the binary classification of susceptibility, meaning that being resistant to one drug indicates being inclined to be resistant to the other(s).

Correlation in the continuous latent MICs can be obtained by modeling their density. When modeling the MIC density, Craig [24] proposed to integrate the uncertainty of the true log_2_MIC values within their underlying intervals to resolve the censorship issue. The author also suggested a Gaussian mixture distribution to reflect the resistant and non-resistant population components without reliance on specific breakpoints. These resistant and non-resistant components of the bacterial populations are indicated by the bimodal distributed frequency plot of the observed MIC. Following this idea, subsequent studies on AMR temporal trends [25] and cross-population correlation in AMR [26] have improved the previous ones that did not address the censored nature or the underlying mixture distribution. Jaspers et al. (2018) [27] developed a Bayesian method to model the joint MIC density of two or more antimicrobials, from which the correlation of type i can be inferred. However, their assumption that the mean MIC of the resistant and non-resistant components was fixed over years could be unrealistic, and inference about correlation of type ii cannot be made from their model.

To our knowledge, research that estimates both types of correlation in antibiotic resistance while accounting for the challenges of analyzing the MIC data has not been conducted yet. In this study, we filled in this gap by modeling for the joint distribution of MIC between antibiotics under a Bayesian framework with a Gaussian mixture model with covariance-dependent mixing weights. Most importantly, the method was able to provide inferences about two types of correlation in antibiotic resistance. Examples of *Salmonella* heidelberg isolates collected by CDC NARMS were analyzed for two pairs of antibiotics, pair 1: amoxicillin-clavulanic acid (AMC) & cephalothin (CEP); and pair 2: ampicillin (AMP) & cephalothin (CEP). These antibiotics are both *β*-lactam antibiotic with ampicillin and amoxicillin-clavulanic acid being in the penicillin class and cephalothin being a cephalosporin, so we would expect both type i and type ii correlation to exist. Our model was assessed by simulation studies to be accurate and robust in estimating the correlations in resistance. The practical contribution of our method is that a large correlation in the continuous latent MIC and/or the binary susceptibility could be used as a way for early detection of MDR phenomenon.

## Methods

### Model notations and assumptions

Our proposed methodology estimates the joint distribution of the antibiotic resistance between several drugs at the isolate level. The MIC data were log-transformed with base 2, which is a commonly seen technique for two-fold serial dilution data. Latent variables of the true log_2_MIC and the component indicator of the isolate were introduced to account for the data censorship and the unobserved components. The joint resistance was assumed to follow a multivariate Gaussian mixture model whose mixing weights contains the conditional dependence between the susceptibilities of the antibiotics. The notations used for model description are as follows:

-
$y_{d,i}^{*}$ : the observed but censored log_2_MIC for isolate *i* tested by drug *d*.
- *y*_*d*,*i*_: the real but latent log_2_MIC for isolate *i* tested by drug *d*.
- *l*_*d*,*i*_, *u*_*d*,*i*_: the lower and upper bound of the real latent value *y*_*d*,*i*_, and *y*_*d*,*i*_ ∈ (*l*_*d*,*i*_, *u*_*d*,*i*_]. The lower and upper bounds for an interval censored log_2_MIC are $y_{d,i}^{*}-1$ and $y_{d,i}^{*}$; for right censored data, $y_{d,i}^{*}$ and +∞; for left censored data, −∞ and $y_{d,i}^{*}$.
- *c*_*d*,*i*_: the latent indicator of the bacterial component from which the isolate *i* tested by drug *d* was drawn. *c*_*d*,*i*_ = 0, 1 indicates the susceptible and resistant component, respectively.

Ranges of the subscripts are *i* = 1, 2, …, *I*, where *I* is the total number of isolates that were tested by multiple antibiotics simultaneously, and *d* = 1, 2, …, *D*, where *D* is the number of antibiotics belonging to different classes that were considered in this research. The supports of the indices remain the same throughout the paper unless otherwise specified.

### Model description

Denote the latent log_2_MIC vector (*y*_1,*i*_, *y*_2,*i*_, …, *y*_*D*,*i*_)^*T*^ of isolate *i* tested by *D* antibiotics simultaneously as ${\vec{y}}_{i}$, the observed outcome as ${\vec{y}}_{i}^{*}$, and the indicator vector (*c*_1,*i*_, *c*_2,*i*_, …, *c*_*D*,*i*_)^*T*^ as ${\vec{c}}_{i}$. The joint antibiotic resistance was assessed at the isolate level as follows. For *i* = 1, 2, …, *I*, and *d* = 1, 2, …, *D*,

$$\begin{array}{c} {\vec{y}}_{i}\overset{ind}{∼}{\text{MVN}}_{D}(\vec{\mu }\left({\vec{c}}_{i}\right),\Sigma ), \end{array}$$ (1)

where the *d*-th element *μ*_*d*_(*c*_*d*,*i*_) of the mean vector $\vec{\mu }\left({\vec{c}}_{i}\right)$ is

$$\begin{array}{c} \mu_{d}\left(c_{d,i}\right)=\{\begin{array}{c} \mu_{0,d},\;\;\text{if}\;c_{d,i}=0\\ \mu_{1,d},\;\;\text{if}\;c_{d,i}=1 \end{array}. \end{array}$$ (2)

The model of latent data assumes that the real log_2_MIC vector for isolate *i* tested by *D* antibiotics follows a *D*-dimensional multivariate normal distribution with a mean vector $\vec{\mu }$ and covariance matrix Σ. The *d*-th element of the mean vector *μ*_*d*_ is dependent on the component *c*_*d*,*i*_ from which isolate *i* was drawn when treated with drug *d*. When considering drug *d* marginally, it is assumed that the classification of isolate *i* follows a Bernoulli distribution with probability *p*_*d*_:

$$\begin{array}{c} c_{d,i}\overset{ind}{∼}\text{Bernoulli}\left(p_{d}\right). \end{array}$$ (3)

Therefore, the mean log_2_MIC of isolate *i* against drug *d* takes value of *μ*_1,*d*_ with probability *p*_*d*_, and takes value *μ*_0,*d*_ with probability 1 − *p*_*d*_. According to the meanings of being susceptible and resistant, the relationship of *μ*_0,*d*_ < *μ*_1,*d*_ should always satisfy for all *d*. Some existing literature argues the phenomenon of antimicrobial multidrug resistance using the proportion of the isolates that are resistant to several antibiotic classes, where the resistant component is defined using the CLSI standards. The resistance classification *c*_*d*,*i*_ in our method is different than the commonly used cut-off method in the sense that the assignment of component here is probabilistic rather than a hard dichotomization.

The covariance matrix Σ contains the variance of the latent log_2_MIC for each drug, regardless of the components. Such a simplification was assumed as it is impossible to accurately estimate variances for both components when there exist large proportions of left and/or right censored data, therefore variance estimation is not the primary goal of our work. The covariance matrix can be decomposed into a correlation matrix (with 1’s on its diagonal and correlation parameters on its off-diagonal, denoted as *R*) sandwiched by the two scale matrices (with the standard deviations on its diagonal and 0’s on its off-diagonal, denoted as *S*). This separation strategy was adopted for the sake of selection of prior distribution for Σ. When considering two drugs (*D* = 2),

$$\begin{array}{c} \begin{array}{cc} \Sigma =(\begin{array}{cc} \sigma_{1}^{2} & \rho \sigma_{1}\sigma_{2}\\ \rho \sigma_{1}\sigma_{2} & \sigma_{2}^{2} \end{array}) & =(\begin{array}{cc} \sigma_{1} & 0\\ 0 & \sigma_{2} \end{array})(\begin{array}{cc} 1 & \rho \\ \rho & 1 \end{array})(\begin{array}{cc} \sigma_{1} & 0\\ 0 & \sigma_{2} \end{array})\\ & ≕SRS. \end{array} \end{array}$$ (4)

Distribution (3) implies independence in the component indicators among isolates for a given antibiotic *d*, which is reasonable. However, independence in the component indicators between antibiotics is not reasonable. When MDR occurs for a certain serotype, we expect not only significantly positive correlation in the latent log_2_MIC, but also conditional dependence in the binary susceptibility classification between different antibiotics. The former can be assessed by parameter *ρ* in the correlation matrix *R*, while the latter can be assessed by introducing the covariance parameter between antibiotic susceptibilities.

To illustrate the approach to modeling the conditional dependence between antibiotic susceptibilities [28], two drugs are given as an example (*D* = 2). Therefore, ${\vec{c}}_{i}\in \Gamma$, where Γ = {{1, 1}, {1, 0}, {0, 1}, {0, 0}}. Denote the covariance between the two antibiotic susceptibilities as *δ*. It can be shown that

$$\begin{array}{c} \begin{array}{cc} P(c_{1,i}=1,c_{2,i}=1) & =p_{1}p_{2}+\delta \\ P(c_{1,i}=1,c_{2,i}=0) & =p_{1}\left(1-p_{2}\right)-\delta \\ P(c_{1,i}=0,c_{2,i}=1) & =\left(1-p_{1}\right)p_{2}-\delta \\ P(c_{1,i}=0,c_{2,i}=0) & =\left(1-p_{1}\right)\left(1-p_{2}\right)+\delta \end{array}. \end{array}$$ (5)

Eq (5), the probabilities of the four components defined by the susceptibilities of two drugs, can be summarised to the following contingency table (Table 1). The feasible range of *δ* is

$$\begin{array}{c} -\text{min}\left(p_{1}p_{2},\left(1-p_{1}\right)\left(1-p_{2}\right)\right)\leq \delta \leq \text{min}\left(p_{1},p_{2}\right)-p_{1}p_{2}. \end{array}$$ (6)

**Table 1:** Contingency table of the components defined by the susceptibilities of two antibiotics.

	*c*_2,*i*_		
		0	1
*c*_1,*i*_	0	(1 − *p*_1_)(1 − *p*_2_) + *δ*	*p*_2_(1 − *p*_1_) − *δ*
	1	*p*_1_(1 − *p*_2_) − *δ*	*p*_1_ *p*_2_ + *δ*

The binary classifications are independent between the two antibiotics when *δ* = 0. Knowing the covariance *δ* is not very straightforward due to its uncommon range, therefore it was transformed to correlation, denoted as *ϕ*, in the binary susceptible classification:

$$\begin{array}{c} \phi =\frac{\delta }{\sqrt{p_{1}\left(1-p_{1}\right)}\sqrt{p_{2}\left(1-p_{2}\right)}}. \end{array}$$ (7)

*ϕ* takes value between -1 and 1. A large *ϕ* implies that if a certain serotype is resistant to one antibiotic, then it is highly likely to be resistant to the other.

The parameter space involved in this Gaussian latent class mixture model is $\Theta =(\vec{\mu },\Sigma ,\vec{p},\delta )$. Let *f*(⋅) be a generic representation for probability density function (pdf) or probability mass function (pmf). Also, denote all latent log_2_MIC values ${\left({\vec{y}}_{1},{\vec{y}}_{2},...,{\vec{y}}_{I}\right)}^{T}$ as **Y**, the corresponding observed values as Y*, and the latent component indicators as **C**. The mean vector of the bivariate normal distribution is $\vec{\mu }={\left(\mu_{1},\mu_{2}\right)}^{T}$, where *μ*_1_ ∈ {*μ*_0,*d* = 1_, *μ*_1,*d* = 1_} and *μ*_2_ ∈ {*μ*_0,*d* = 2_, *μ*_1,*d* = 2_}. The marginal probabilities of being resistant form a vector $\vec{p}={\left(p_{1},p_{2}\right)}^{T}$. Then under such a context of two antibiotics, the data likelihood can be written as

$$\begin{array}{c} \begin{array}{cc} f(Y^{*}|\Theta ) & =\prod_{i=1}^{I}f\left({\vec{y}}_{i}^{*}|\Theta \right)\\ & =\prod_{i=1}^{I}\int_{l_{2,i}}^{u_{2,i}}\int_{l_{1,i}}^{u_{1,i}}\sum_{{\vec{c}}_{i}\in \Gamma }f\left({\vec{y}}_{i},{\vec{c}}_{i}|\Theta \right)dy_{1,i}dy_{2,i}\\ & =\prod_{i=1}^{I}\int_{l_{2,i}}^{u_{2,i}}\int_{l_{1,i}}^{u_{1,i}}\sum_{{\vec{c}}_{i}\in \Gamma }f\left({\vec{y}}_{i}|\vec{\mu },\Sigma ,{\vec{c}}_{i}\right)\cdot f\left({\vec{c}}_{i}|\vec{p},\delta \right)dy_{1,i}dy_{2,i}. \end{array} \end{array}$$ (8)

In (8), the first equality is due to independence among the submitted isolates. The second equality is to sum the latent component indicators over their possible values, and to integrate the continuous log_2_MIC over the intervals of which the discrete measurements lay in. The two parts in $\sum_{{\vec{c}}_{i}\in \Gamma }f\left({\vec{y}}_{i}|\vec{\mu },\Sigma ,{\vec{c}}_{i}\right)\cdot f\left({\vec{c}}_{i}|\vec{p},\delta \right)$ are defined by the bivariate normal distribution (1) and the contingency Table 1, which forms a Gaussian mixture distribution with mixing weights containing the covariance between the antibiotic susceptibility. Consequently, a Bayesian model is obtained:

$$\begin{array}{c} \begin{array}{cc} f(\Theta ,Y,C|Y^{*}) & =f\left(\vec{\mu },\Sigma ,\vec{p},\delta ,Y,C|Y^{*}\right)\propto f\left(Y^{*},Y,C,\vec{\mu },\Sigma ,\vec{p},\delta \right)\\ & =f\left(Y^{*}|Y,C,\vec{\mu },\Sigma ,\vec{p},\delta \right)\cdot f\left(Y,C|\vec{\mu },\Sigma ,\vec{p},\delta \right)\cdot f\left(\vec{\mu },\Sigma ,\vec{p},\delta \right)\\ & =f\left(Y^{*}|Y,C,\vec{\mu },\Sigma ,\vec{p},\delta \right)\cdot f\left(Y,C|\vec{\mu },\Sigma ,\vec{p},\delta \right)\cdot f\left(\vec{\mu }\right)\cdot f\left(\Sigma \right)\cdot f\left(\vec{p}\right)\cdot f\left(\delta \right) \end{array} \end{array}$$ (9)

In (9), the pmf $f(Y^{*}|Y,C,\vec{\mu },\Sigma ,\vec{p},\delta )$ associates Y*, the observed log_2_MIC, with its underlying latent values **Y**, and corresponds to the lowest hierarchy of the model. The term $f\left(Y^{*}|Y,C,\vec{\mu },\Sigma ,\vec{p},\delta \right)\cdot f\left(Y,C|\vec{\mu },\Sigma ,\vec{p},\delta \right)$ is expressed in Eq (8). Selection of the prior distributions for the model parameters is described in the following Subsection of Prior specification.

### Prior specification

A joint prior distribution of the unknown model parameters $\Theta =(\vec{\mu },\Sigma ,\vec{p},\delta )$ is required by the full Bayesian analysis. If assuming independence, it can be expressed as the product of each individual prior distribution as shown in the last equality in (9). Diffuse Gaussian priors were selected for $\vec{\mu }$ due to the *a priori* lack of knowledge about the means: *μ*_0,1_, *μ*_1,1_, *μ*_0,2_, *μ*_1,2_ ∼ *N*(0, 10000). The constraint that *μ*_0,*d*_ < *μ*_1,*d*_ was applied to reflect the relationship between the susceptible and resistant component. Similarly, a non-informative prior distribution Beta(0.5, 0.5) was determined for *p*_1_ and *p*_2_. A default uniform prior was assigned to the covariance parameter *δ* on its feasible range: *f*(*δ*) ∝ 1 for −min(*p*_1_ *p*_2_, (1 − *p*_1_)(1 − *p*_2_)) ≤ *δ* ≤ min(*p*_1_, *p*_2_) − *p*_1_ *p*_2_.

As for the prior distribution of the covariance matrix Σ, since the widely-used inverse Wishart distribution tends to bias downward the posterior correlation [29], the separation strategy [30] was adopted instead. According to the recommendation of the Stan development team [31], an LKJ prior was determined for the correlation matrix [32]: *f*(*R*) ∝ |*R*|^*ν*−1^, *ν* > 0. By choosing LKJ (*ν* = 1) for the correlation matrix *R*, the density of its prior is uniform over the correlation matrices of dimension *D* = 2. We assigned weakly informative half-Cauchy priors to the scale parameters: *σ*_1_, *σ*_2_ ∼ Cauchy^+^(0, 2).

### Alternative approaches to assess correlation

It is often of interest to compare a new approach with alternative methods. As a comparison to the Bayesian approach to estimating the correlation in latent log_2_MIC and in susceptibility, alternative methods [20, 21] were brought up and used for comparison. Two alternative approaches for the correlation related to MIC (type i correlation referred to above) are Pearson correlation and Spearman rank correlation. We used Spearman rank correlation for the observed log_2_MIC because the rank approach acknowledges the ordinal nature of the MIC. For the binary correlation (i.e. type ii), the Spearman and Pearson methods give the same result—that is they calculate the correlation in the binary classification expressed as *c*_*d*,*i*_ in the model—as the estimate using the GEE approach [22].

## Real data analyses

### Data description

The dataset used to apply the Bayesian approach to modeling the MDR is the human population of NARMS, which was launched in 1996 within the framework of CDC’s Emerging Infections Program and the Foodborne Diseases Active Surveillance Network (FoodNet). Participating public health laboratories submit their every 20th non-typhoidal *Salmonella*, *Campylobacter*, *Shigella*, and *Escherichia coli* O157 isolates to CDC for antibiotic susceptibility testing [33]. The data were published with year of collection, genus, serotype, MIC value tested against multiple antibiotics, the test conclusion (resistant or not), etc. Currently, CDC NARMS routinely tests for susceptibility to 18 antibiotic agents that belong to 12 classes of drugs.

**Fig 2:**
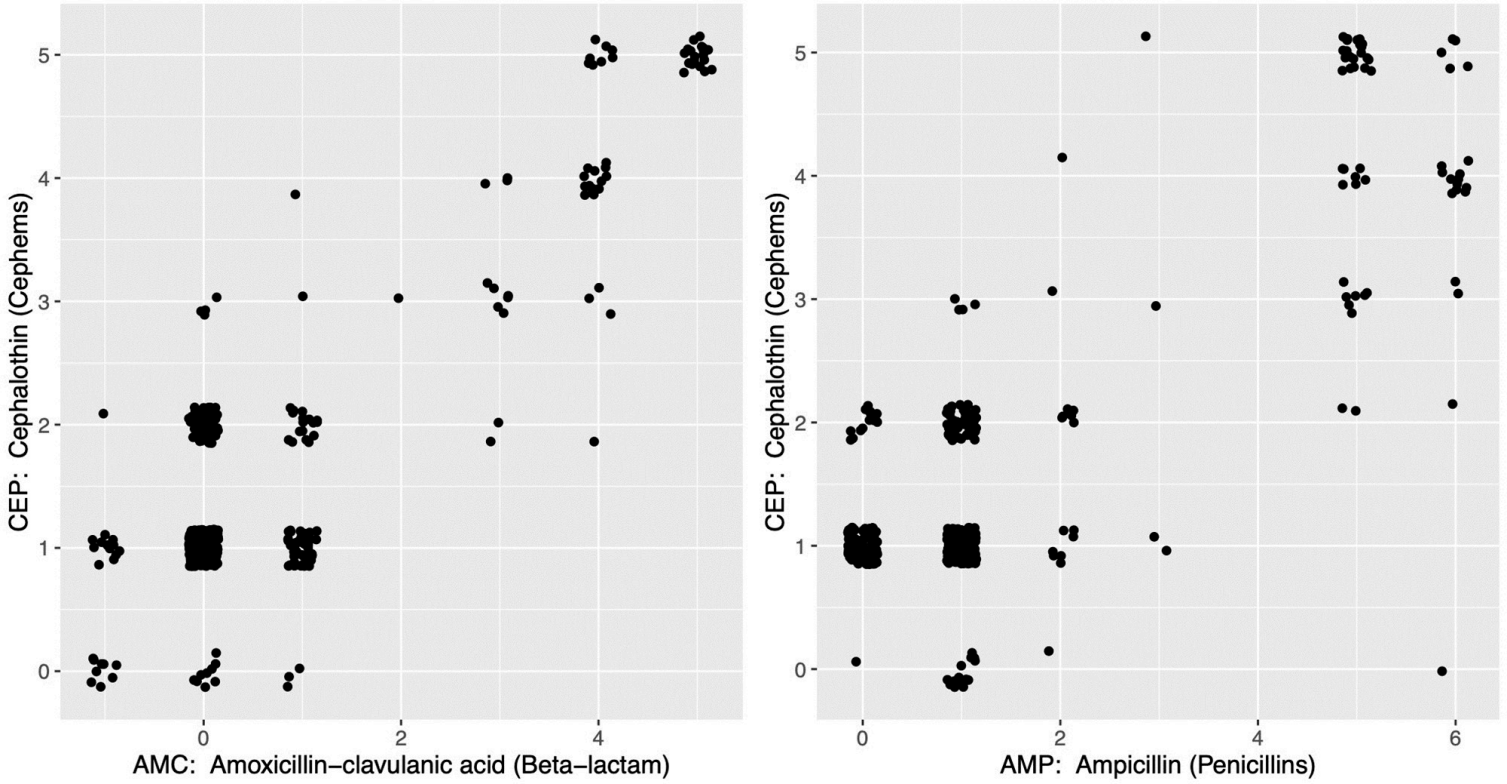
Scatter plots of the observed log_2_MIC (MIC in unit μg/mL) with jittering of *Salmonella* heidelberg isolates tested by AMC & CEP (left) and AMP & CEP (right) in NARMS human population during 1996-2003.

We limited our analyses to an important *Salmonella* serotype: *S.* heidelberg, which has 1196 isolates, accounting for 3.12% of the total 38311 *Salmonella* enterica isolates. The pairs of antibiotics we focused on are AMC (Amoxicillin-clavulanic acid, *β*–lactam combination agent class) & CEP (Cephalothin, Cephalosporin class) and AMP (Ampicillin, Penicillin class) & CEP. Since the susceptibility of heidelberg to CEP was not tested after 2003, there were 498 isolates recorded for each pair. The scatter plots of the observed log_2_MIC of the two pairs are shown in Fig 2. The CLSI cut-off value after log_2_ transformation is 5 for all drugs studied here. Thus, there were 3.82% isolates jointly resistant to AMC & CEP, and 5.22% jointly resistant to AMP & CEP.

### Implementation

A Bayesian analysis of Markov Chain Monte Carlo (MCMC) was implemented in R version 3.6.3 using the *rstan* package [34] in order to draw inference from the posterior distribution *f*(Θ, **Y**, **C**|Y*) which has no closed form. The sampling algorithm adopted was the No-U-Turn sampler variant of Hamiltonian Monte Carlo which is efficient, and hence the preferred method in Stan [35].

For each of the two example pairs, the initial values were determined based upon the log_2_MIC observations to avoid random initialization. More specifically, the mean vector $\vec{\mu }$ was obtained by taking the arithmetic mean of the censored observations within each component for each drug. The standard deviations in the scale matrix *S* were each calculated directly from the observations of each drug. The Spearman rank correlation of the observations between the paired drugs was used as the initial value of type i correlation *ρ*. The proportions of the resistant isolates were regarded as the initial values of $\vec{p}$. The difference between the jointly resistant proportion and the product of the marginal resistant proportions is used as the initial value for type ii covariance *δ*. Here, the resistant isolates are classified by CLSI standards.

The model implementation was based on three chains with 10,000 MCMC iterations, disregarding the first 50% realizations as the burn-in session. The MCMC convergence was examined with the Gelman–Rubin diagnostic [36]. The point estimates and the 95% credible intervals of the model parameters were obtained by taking the means and the 2.5th and 97.5th percentiles of the posterior draws after the burn-in portion. The application results were summarized and can be found in the following subsection. Model specification, implementation, and other relevant R scripts are available in a public GitHub repository (GitHub: https://github.com/MinZhang95/AMR-MDR).

### Application results

The correlations in both the continuous latent log_2_MIC and the binary susceptibility are of particular interest when modeling the joint distribution of resistance to several antibiotics. Their estimation results can be found in Table 2, where the point estimation, standard deviation, and credible interval for the two parameters of each example pair are presented.

**Table 2:** Estimations of the correlation in the latent log_2_MIC (*ρ*) and correlation in susceptibility classification (*ϕ*) using *Salmonella* heidelberg isolates of NARMS human population tested by AMC & CEP and AMP & CEP during 1996-2003.

Pair	Parameter	Estimation	Standard deviation	Credible interval
AMC & CEP	*ρ*	0.6303	0.0450	(0.5352, 0.7115)
	*ϕ*	0.9677	0.0188	(0.9210, 0.9935)
AMP & CEP	*ρ*	0.2818	0.0934	(0.0884, 0.4628)
	*ϕ*	0.9174	0.0295	(0.8524, 0.9663)

Since the credible interval for each correlation parameter of each case is above 0, we are able to conclude significant correlations. In other words, the correlations in the latent log_2_MIC and in the binary susceptibility are significantly positive for the paired antibiotics. The estimations in the correlation of binary susceptibility *ϕ* are especially high, with values greater than 0.9 for both cases. The point estimations of *ρ* and *ϕ* from the Spearman correlation are 0.3986 and 0.9042 for AMC & CEP, 0.1316 and 0.6381 for AMP & CEP, which has some discrepancy from the Bayesian estimations. To assess the performance of the hierarchical Bayesian model, with the Spearman correlation as a comparison, simulation studies were conducted.

## Simulation

To assess the performance of the proposed Bayesian approach to estimating the correlation in antibiotic resistance, three simulation studies were conducted using the estimated results from the two application examples in the Real data analyses Section and a negative control as the underlying data generators. The negative control shared the same parameters as the application results of AMC & CEP except that it assumed null correlations between the two antibiotics. Greek letters with “hats” on top are adopted to denote the known model parameters $\hat{\Theta }=(\hat{\vec{\mu }},\hat{\Sigma },\hat{\vec{p}},\hat{\delta })$.

To simulate *I* = 498 isolates (which is the size of the real data examples):

1. Generate *I* categorical realizations following the multinomial distribution and denote the counts within each category as ${\left(n_{0,0},n_{0,1},n_{1,0},n_{1,1}\right)}^{T}\overset{}{∼}\text{Multinomial}\left(I,\hat{P}\left(c_{1,i}=0,c_{2,i}=0\right),\hat{P}\left(c_{1,i}=0,c_{2,i}=1\right),\hat{P}\left(c_{1,i}=1,c_{2,i}=0\right),\hat{P}\left(c_{1,i}=1,c_{2,i}=1\right)\right)$. Each $\hat{P}\left(\cdot \right)$ is calculated by entering $\hat{\vec{p}}$ and $\hat{\delta }$ into the probabilities shown in the contingency Table 1.
2. Generate **Y**, the latent log_2_MIC, which are composed of
  *n*_0,0_ data from ${\text{MVN}}_{2}((\begin{array}{c} {\hat{\mu }}_{0,1}\\ {\hat{\mu }}_{0,2} \end{array}),\hat{\Sigma })$,*n*_0,1_ data from ${\text{MVN}}_{2}((\begin{array}{c} {\hat{\mu }}_{0,1}\\ {\hat{\mu }}_{1,2} \end{array}),\hat{\Sigma })$, *n*_1,0_ data from ${\text{MVN}}_{2}((\begin{array}{c} {\hat{\mu }}_{1,1}\\ {\hat{\mu }}_{0,2} \end{array}),\hat{\Sigma })$,*n*_1,1_ data from ${\text{MVN}}_{2}((\begin{array}{c} {\hat{\mu }}_{1,1}\\ {\hat{\mu }}_{1,2} \end{array}),\hat{\Sigma })$.
3. Convert the latent log_2_MIC to the censored Y*: $y_{d,i}^{*}=\{\begin{array}{c} \text{lowest}\;\text{concentration},y_{d,i}\leq \text{lowest}\;\text{concentration}\\ ]y_{d,i},\text{lowest}\;<y_{d,i}\leq \text{highest}\;\text{concentration}\\ \text{highest}\;\text{concentration},y_{d,i}>\text{highest}\;\text{concentration} \end{array}$; ⌈⋅⌉ represents the ceiling of a number.
  The lowest and highest concentrations refer to the two extremes of the dilution range, which is drug-dependent. Each of the three antibiotics studied here has its own spectrum of serial dilution.
4. Assign the binary conclusions (non-resistant v.s. resistant) according to the censored data using the CLSI breakpoints. The censored log_2_MIC data simulated from the above steps are considered as susceptible if below the breakpoint which is 5; otherwise, classified as resistant.

We obtained *n* = 100 simulated datasets by repeating the above procedure 100 times for each case. Both the proposed Bayesian approach and the Spearman methods were applied to the simulated datasets. Therefore, 100 estimation results were attained for each pair. Since we are interested in comparing the accuracy and robustness of the two methods, the mean absolute error (MAE) and the root of mean squared error (RMSE) were calculated for each set of results, as displayed in Table 3. Scenarios 1-3 represent data generators of applications AMC & CEP, AMP & CEP, and the null case, respectively.

**Table 3:** Comparisons of the estimated correlations for latent log_2_MIC (*ρ*) and binary classification (*ϕ*) between the Bayesian and Spearman method through simulations.

Scenario	Parameter	Truth	Method	Estimation	SD	MAE	RMSE
Scenario 1	*ρ*	0.6303	Bayesian	0.6304	0.0409	0.0331	0.0407
			Spearman	0.7040	0.0388	0.0756	0.0832
	*ϕ*	0.9677	Bayesian	0.9461	0.0174	0.0228	0.0276
			Spearman	0.6302	0.0746	0.3375	0.3455
Scenario 2	*ρ*	0.2818	Bayesian	0.2649	0.0655	0.0522	0.0671
			Spearman	0.1933	0.0472	0.0892	0.1001
	*ϕ*	0.9174	Bayesian	0.8926	0.0218	0.0269	0.0329
			Spearman	0.7231	0.0493	0.1943	0.2004
Scenario 3	*ρ*	0.0000	Bayesian	-0.0074	0.0590	0.0478	0.0592
			Spearman	-0.0096	0.0481	0.0390	0.0489
	*ϕ*	0.0000	Bayesian	0.0174	0.0416	0.0344	0.0449
			Spearman	0.0055	0.0459	0.0370	0.0460

## Discussion and conclusion

In this work, we proposed a Bayesian latent class mixture model as a tool to assist with assessment of the antibiotic multidrug resistance phenomenon. When MDR occurs, we expect strong correlation in the latent log_2_MIC between antibiotics, and also strong correlation in the binary susceptibility classification. This Bayesian approach is capable of addressing both characteristics while modeling the resistance level for multiple drugs. The approach offers the opportunity to obtain more from the MIC data than is currently being extracted. On the same dataset, using the proposed model, researchers and public health officials are able to (a) assess the correlation of MIC for antibiotics even below the resistant threshold; (b) assess the binary classification of organisms resistant or non-resistance in a manner independent of the pre-determined threshold values; and (c) account for the censored nature of the MIC data.

One of the goals of the surveillance programs is to early detect issues with the hope that interventions can mitigate risk. The purpose of correlation estimation is not merely to test for significant correlations, but also to identify newer correlations that are emerging. The question could be “Are two antibiotics that were previously not correlated starting to show positive correlations?”. And the answers inferred from our approach could trigger an investigation into the mechanism of that correlation. Similarly, if the correlation between two classes of antibiotic increases, this might also warrant an investigation. As such, we envision that most of the findings from our analyses will be hypotheses generating. Further, the presence of correlation should be combined with other information available in the dataset. In our examples, only 3.82% of isolates are jointly resistant to AMC & CEP, and 5.22% jointly resistant to AMP & CEP, so concerns about correlation might be tempered by the low resistant prevalence. Public health officials would need to balance the three pieces of information: type i correlation, type ii correlation and resistant prevalence when making decisions.

The currently used analytical methods, Spearman correlation in log_2_MIC and the binary conclusions, were compared against the proposed model in Table 3. What can be seen from the bias that arises is that although the Spearman calculation is straightforward, it does not consider the censored nature of the MIC data, or the mixture structure of susceptible and resistant isolates. As shown in the simulation results in Table 3, when correlations exist, the advantages in the Bayesian model are obvious in terms of the mean absolute error (MAE) and the root of mean squared error (RMSE), meaning that the Bayesian method provides a more accurate and robust estimation of the correlations. When correlations do not exist, such advantages become less obvious and the two methods perform comparably well. It can be found that the alternative method tends to underestimate the correlation in the susceptibility status when the ground truth is not null, which could be misleading in real life. Though the Spearman correlation of 0.6302 in the binary conclusions for the AMC & CEP example is quite high, it is not as close to the truth as the Bayesian estimation. However, the proposed model does require a mixture of susceptible and resistant components in order to obtain an accurate estimation, and we found this to be true for many (but not all) of the organism-antibiotic combinations in the NARMS datasets.

The proposed Bayesian model was articulated for the two dimensional case where two antibiotics were considered simultaneously. This work can be generated to three or higher dimensions and applied to more antibiotics. Application of this model to three drugs and more could promote our understanding to the surveillance data and serve as an alert when MDR emerges.
